# Neuroligin-1 knockdown reduces survival of adult-generated newborn hippocampal neurons

**DOI:** 10.3389/fnins.2014.00071

**Published:** 2014-04-10

**Authors:** Eric Schnell, Thomas H. Long, AeSoon L. Bensen, Eric K. Washburn, Gary L. Westbrook

**Affiliations:** ^1^Portland VA Medical CenterPortland, OR, USA; ^2^Department of Anesthesiology and Perioperative Medicine, Oregon Health and Science UniversityPortland, OR, USA; ^3^School of Medicine, Oregon Health and Science UniversityPortland, OR, USA; ^4^The Vollum Institute, Oregon Health and Science UniversityPortland, OR, USA

**Keywords:** adult neurogenesis, neuroligin-1, cell survival, dendritic spine, synaptogenesis, dendritic growth

## Abstract

Survival of adult-born hippocampal granule cells is modulated by neural activity, and thought to be enhanced by excitatory synaptic signaling. Here, we report that a reduction in the synaptogenic protein neuroligin-1 in adult-born neurons *in vivo* decreased their survival, but surprisingly, this effect was independent of changes in excitatory synaptic function. Instead, the decreased survival was associated with unexpected changes in dendrite and spine morphology during granule cell maturation, suggesting a link between cell growth and survival.

## Introduction

Adult-born hippocampal granule cells are generated throughout mammalian life from stem cells residing in the hippocampal subgranular zone (Taupin and Gage, 2002). During the first 4 weeks after mitosis, newborn granule cells extend dendrites and receive synaptic input from GABAergic interneurons and excitatory afferents (Esposito et al., 2005) in a process important for learning and memory (Ming and Song, 2011). However, between weeks 1 and 4, the number of newborn granule cells decreases by about 50% as a result of apoptosis (Kempermann et al., 2003; Sierra et al., 2010), subject to an array of environmental and biological influences. For example, both active learning and environmental enrichment increase survival (Kempermann et al., 1997; Gould et al., 1999). As these conditions also increase dendritic arborization and dendritic spine formation (van Praag et al., 2000; Ambrogini et al., 2010; Tronel et al., 2010), it has been hypothesized that learning enhances the synaptic integration of newborn cells, providing a survival signal for cells that might otherwise be relegated to an apoptotic fate (Gould et al., 1999). Consistent with this idea, synaptic NMDARs are necessary for newborn granule cell survival (Tashiro et al., 2006) and activity-dependent spine formation (Engert and Bonhoeffer, 1999).

The synaptogenic protein neuroligin-1 has a critical role in functional excitatory synapse formation (Craig and Kang, 2007; Sudhof, 2008), and neuroligin-1 overexpression in adult-born granule cells *in vivo* can drive excitatory synapse formation and enhance dendritic process outgrowth (Schnell et al., 2012). Neuroligin-1 knockdown reduces excitatory synapse number in mature dentate granule cells (Shipman and Nicoll, 2012), but the role of endogenous neuroligin-1 in immature adult-born cells during their synaptic integration into the dentate gyrus is unknown. Here, we use reduce neuroligin-1 expression during the maturation of adult-born granule cells *in vivo*. We find that endogenous neuroligin-1 has a critical role in dictating the morphology and survival of adult born granule cells, and that these roles are independent of functional synaptogenesis.

## Materials and methods

### Retrovirus production

Newborn granule cells were identified using Moloney Murine Leukemia Virus-based retroviral vectors, which require mitosis for cell transduction and are used to label and manipulate adult-born granule cells (van Praag et al., 2002). GFP- and mCherry-expressing retroviruses were created using a pSie-based viral backbone, with fluorophore expression driven by a ubiquitin promoter and followed by a woodchuck post-transcriptional regulatory element (Luikart et al., 2011). Knockdown was achieved using two copies of a validated neuroligin-1 hairpin shRNA sequence (GGAAGGTACTGGAAATCTATTCAAGAGATAGATTTCCAGTACC TTCCTTTTTT; Chih et al., 2005; Shipman et al., 2011) separately driven by H1 and U6 promoters, as this provided the highest degree of knockdown in a separate set of experiments (B. Luikart, pers. commun.). To validate our shRNA knockdown construct, we transfected HEK cells with a plasmid encoding HA-tagged mouse neuroligin-1 together with either our retroviral shRNA plasmid or a retroviral control vector, and verified that our shRNA construct reduced neuroligin-1 mRNA levels using quantitative PCR (% knockdown = 74.9 ± 9.3%, *n* = 3 independent transfections). Viral particles were generated as previously described (Schnell et al., 2012).

### Injection

All procedures were performed in accordance with OHSU and VA IACUC-approved protocols. Male and female C57Bl/6 mice were injected with retroviral particles at 6–8 weeks of age, and littermates were used for injections of control and knockdown viruses. Mice were anesthetized using inhaled isoflurane while continuously monitored on a heated pad. After shaving the scalp, a midline skin incision was made, and skin was retracted to expose the skull. Bilateral craniotomies were made using a stereotaxic drill, and 2 μl viral concentrate was injected into each dentate gyrus (relative to bregma: *x* = ±1.1 mm, *y* = −1.1 mm, *z* = −2.5, −2.3 mm) using a Stoelting QSI injector driving a Hamilton syringe. The scalp was closed, lidocaine jelly applied, and animals recovered in a warm chamber before returning to their home cage. Prophylactic oral acetaminophen was administered for 2 days.

### Confocal imaging

At 1–4 weeks after injection, mice were euthanized with an i.p. injection of avertin, followed by transcardiac perfusion with PBS containing 4% PFA and 4% sucrose. Brains were post-fixed overnight prior to sectioning (100 μm), and stained to enhance fluorescent protein detection (Invitrogen anti-GFP Alexa488 conjugate, 1:400; Clontech anti-mCherry 1:2000 followed by an Alexa568 secondary). Slides were coded after mounting and blindly imaged using a Zeiss LSM 780 microscope. For analysis of dendritic branching patterns, intact granule cells were imaged with a 40 × 1.2NA objective using a Z-stack to cover the entire extent of the dendritic tree. Cells were subsequently traced in three dimensions, flattened, and measured using ImageJ software. For spine quantification, 2–3 linear stretches of dendrite from the middle molecular layer were imaged for each cell using a 40 × 1.4NA objective, and manually counted off-line. Although not explicitly quantified, dendritic spine density was roughly similar for any individual cell throughout all layers of the molecular layer (inner, middle, and outer), and the middle was quantified for consistency. Sholl analyses of dendritic branching were performed using ImageJ with a Sholl plug-in (Ghosh lab), and statistical significance at each interval was determined using a Two-Way repeated measures ANOVA followed by a *post-hoc* Bonferroni correction. Spine head cross sectional area was determined by measuring the length and width of each spine head from a confocal image stack, and using the equation *area* = (*length* * *width*) * π /4, which gives an excellent approximation of the spine head cross-sectional area as previously determined (Zhao et al., 2006). Spine head areas were compared between groups by Kolmogorov–Smirnov test (Prism). All images were acquired, quantified, and analyzed by an experimenter blind to experimental condition. Data was compared between groups using unpaired two-way *t*-tests, with data expressed as mean ± s.e.m. Each sample (each n) contributing to the mean represents the summary data from a single cell.

### Cell survival assay

To study cell survival, groups of mice were injected with a 1:1 mixture of two viruses as previously described (Tashiro et al., 2006). The mCherry-expressing virus controlled for viral injection coordinates and baseline cell survival, and the GFP-expressing virus contained either a vector-only (control) or the neuroligin-1 knockdown shRNA. Each dentate received 4 μl mixed viral stock (2 μl of each virus), with 6–8 animals injected per condition for each time interval. Each virus came from a single viral preparation, keeping relative viral titers constant for each viral pair. From each animal, six 100 μm coronal slices near the injection site were stained and imaged in both red and green channels. Infected cells were counted off-line by an experimenter blind to experimental condition, and GFP+ and mCherry+ cells were counted independently without regards to colocalization. The number of GFP-expressing cells was divided by number of mCherry expressing cells for each animal, and this ratio was normalized to the average (± s.e.m.) ratio of green to red cells at 7 days for each virus pair, to control for the small differences in viral titer between the two co-injected viruses.

### Electrophysiology

Acute hippocampal slices were prepared from virus-injected animals 21 days after injection (Schnell et al., 2012). Whole cell recordings were obtained from GFP-positive granule cells using combined fluorescence and differential interference contrast microscopy. Cells were voltage clamped at −70 mV using an Axopatch 200B amplifier; the Cs-gluconate based internal solution contained (in mM): 100 gluconic acid, 10 EGTA, 10 HEPES, 17.5 CsCl, 8 NaCl, 2 Mg-ATP, 0.3 Na-GTP, pH = 7.3 (using 50% CsOH), 290 mOsm. The external solution contained (in mM): 125 NaCl, 25 NaHCO_3_, 2.5 KCl, 1.25 NaH_2_PO_4_, 2.0 CaCl_2_, 1.0 MgCl_2_, 25 D-glucose,and 0.01 SR95531, bubbled with 95% O_2_–5% CO_2_. Spontaneous EPSCs (sEPSCs) were obtained from at least 5 min of consecutive recording for each cell; mEPSCs were obtained by 5 min of consecutive recording in the presence of 1 μM TTX. Traces were acquired at 10 kHz and off-line filtered at 2 kHz prior to analysis. mEPSCs and sEPSCs were automatically detected using a template-matching algorithm (Axograph), and manually accepted/rejected by an experimenter blinded to the experimental condition. Evoked AMPA receptor and combined AMPAR and NMDAR events were recorded after stimulation of the middle molecular layer using a bipolar electrode (FHC), while holding the cell membrane voltage at −70 and +40 mV, respectively. The NMDAR component of the EPSC was quantified as the amplitude of the current recorded 60 ms after the stimulus. Paired pulse facilitation was assessed to assay presynaptic release probability (Zucker and Regehr, 2002). Two identical stimuli were delivered via the bipolar electrode with inter-stimulus intervals of 50–250 ms, and measurements were taken from an average of 10 trials for each time interval for each cell. Series resistance was measured on-line and cells were discarded if this changed by >20% during the course of the experiment. In recordings from visually identified 21-day-old granule cells, passive membrane properties did not differ between the two groups of cells (input resistance: control = 1282 ± 183 mΩ, shNL1 = 2121 ± 526 mΩ, *p* > 0.1; capacitance: control = 27.2 ± 1.2 pF, shNL1 = 25.5 ± 1.3 pF, *p* > 0.3; *n* = 27, 26 cells). Summary data are presented as the mean value ± s.e.m. for each parameter, with n = the number of cells per condition, and were compared using unpaired two-tailed *t*-tests.

## Results

### Neuroligin-1 knockdown reduces the formation of dendritic spines in newborn cells

**Figure 1:**
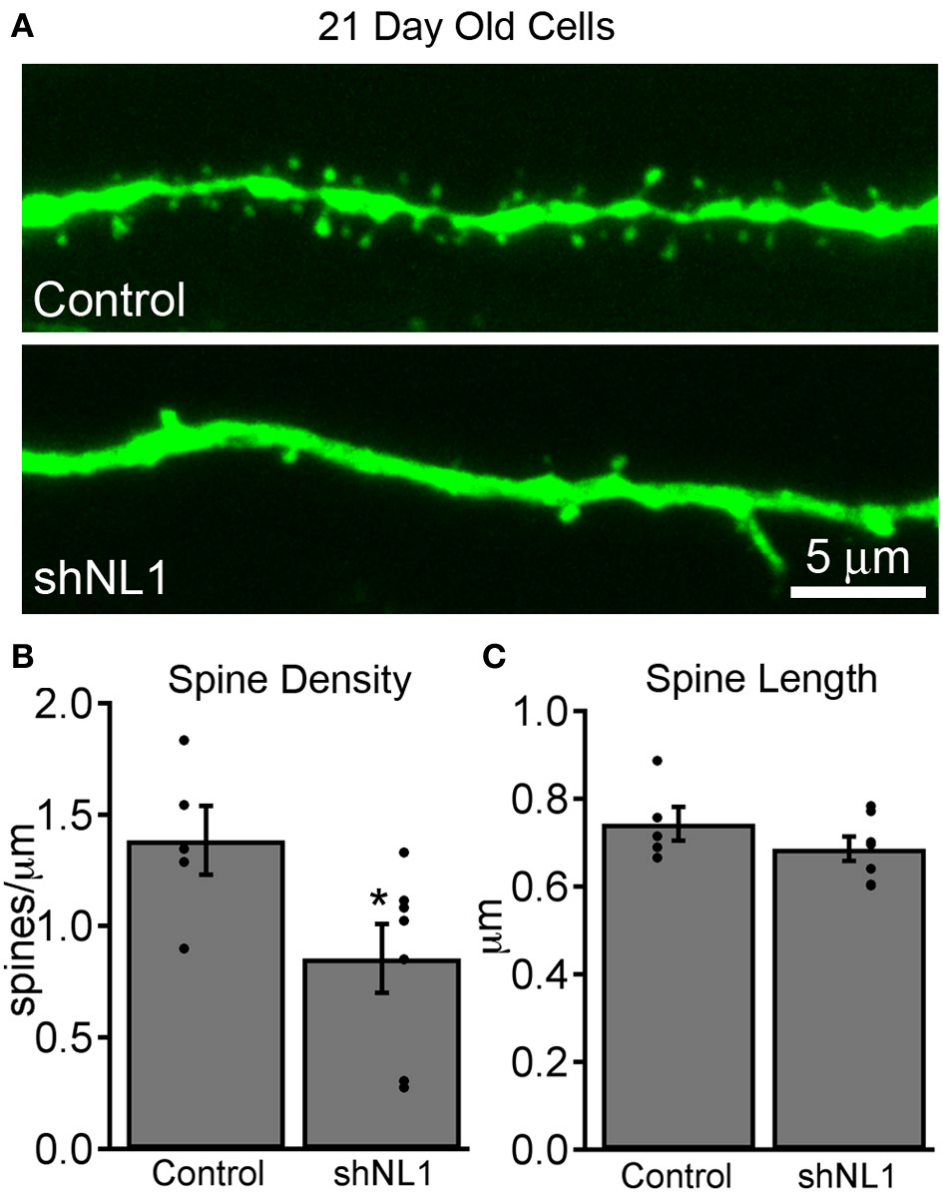
**Neuroligin-1 knockdown reduces dendritic spine density in immature adult-born granule cells. (A)** Representative dendritic segments from retroviral vector control (top) and neuroligin-1 shRNA injected cells (shNL1; bottom) 21 days after mitosis. **(B)** Summary graph of dendritic spine density in the dentate middle molecular layer for each condition (control, *n* = 5; shNL1, *n* = 7; ^*^p < 0.05), with individual data points plotted for each condition. **(C)** Neuroligin-1 knockdown does not alter dendritic spine length (*p* > 0.2).

Newborn granule cells were identified using retroviral vectors, which require mitosis for cell transduction and are used to label and manipulate adult-born granule cells (van Praag et al., 2002). We injected shRNA-encoding retroviral particles into adult mouse hippocampal dentate gyrus to reduce neuroligin-1 levels in adult-born granule cells *in vivo*, and used co-expressed GFP to identify and characterize cells at varying post-mitotic intervals. As expected based on its role in excitatory synaptogenesis (Chih et al., 2005), neuroligin-1 knockdown during adult-born granule cell maturation decreased dendritic spine density at 21 days post-mitosis (Figures 1A,B), without a change in average spine length (Figure 1C).

**Figure 2:**
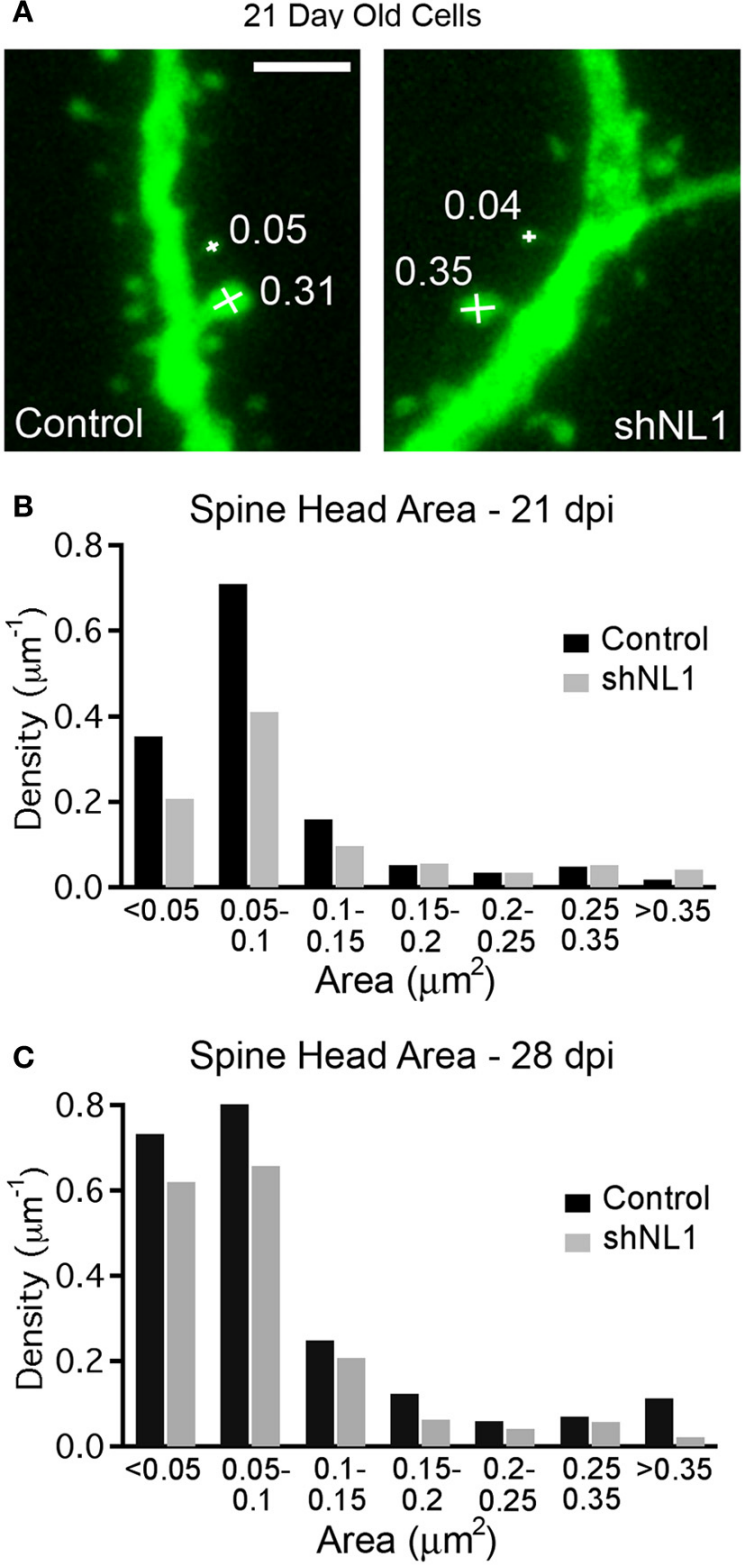
**Neuroligin-1 knockdown reduces the density of filopodial spines in 21-day-old adult-born granule cells. (A)** Spine head area measurement demonstrating measurement technique (see methods) with spine head areas denoted for selected spines (in μm^2^). Scale 2 μm. **(B)** Histogram of spine density (spines per μm dendrite length) at 21 days post-mitosis for each condition, demonstrating a selective decrease in the density of small spines/filopodia (*p* < 0.05, K–S test). **(C)** Histogram of spine density (spines per μm dendrite length) at 28 days post-mitosis for each condition (*p* < 0.05, K–S test).

**Figure 3:**
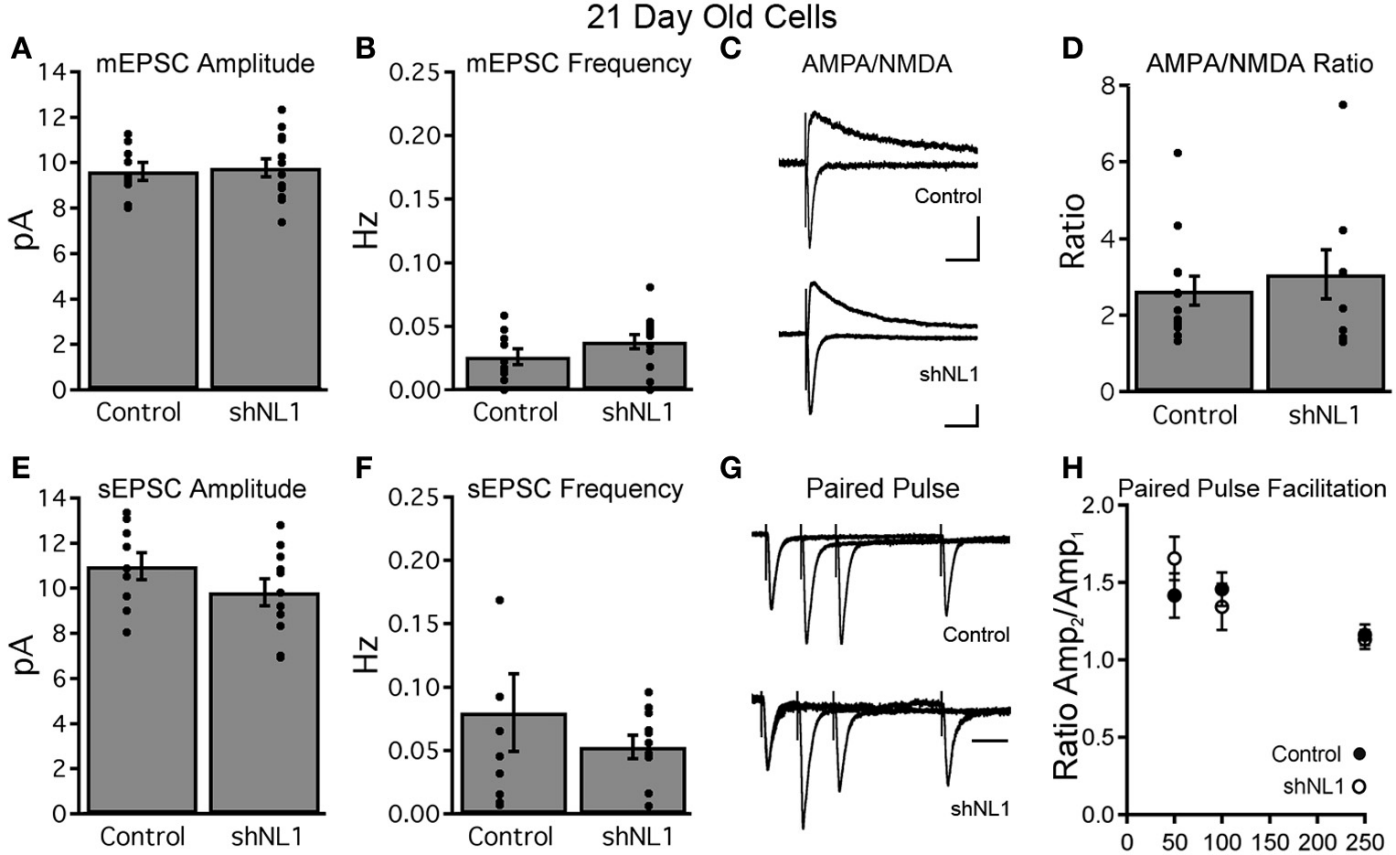
**Neuroligin-1 knockdown does not affect basal synaptic transmission in 21-day-old neurons. (A,B)** Miniature EPSC recordings from retrovirally labeled control and neuroligin-1 knockdown neurons demonstrate no difference in mEPSC amplitude **(A)** or frequency **(B)** between groups (control, *n* = 10 cells; shNL1, *n* = 16 cells; *p* > 0.1). **(C)** Representative evoked EPSCs from labeled granule cells demonstrated similar AMPAR-meditated and combined AMPAR/NMDAR responses at −70 and +40 mV, respectively, suggesting that 21 day-old shNL1 cells did not selectively lose NMDAR-only synapses. Artifacts truncated for clarity. Scale 20 pA, 50 ms. **(D)** Summary data demonstrated no change in the AMPA/NMDA ratio (control, *n* = 13 cells; shNL1, *n* = 9 cells; *p* > 0.5). **(E,F)** EPSC recordings in the absence of TTX showed no difference in sEPSC amplitude **(E)** or frequency **(F)** between groups (control, *n* = 9 cells; shNL1, *n* = 11 cells; *p* > 0.1). **(G)** Representative evoked EPSC (peak scaled to the first EPSC) demonstrate similar paired pulse facilitation between groups, suggesting no alteration in presynaptic properties. Scale 50 ms. **(H)** Summary data demonstrated no change in the paired pulse ratio (control, *n* = 10 cells; shNL1, *n* = 6 cells; *p* > 0.2 each interval).

Immature granule cells are innervated by a mixture of axospinous and axodendritic (shaft) synapses, and also form multiple small, non-synaptic filopodia (Toni et al., 2007), which presumably represent precursors to synapse formation. Interestingly, measurements of dendritic spine head cross-sectional area demonstrated that the reduction in spine density was due to fewer small filopodia-like spines without a change in large, mushroom-shaped spines (Figures 2A,B; *p* < 0.05), the latter of which constitute a minority of spine-like structures at this stage (Zhao et al., 2006). Consistent with a preponderance of non-synaptic filopodia in immature adult-born neurons (Toni et al., 2007), we failed to find alterations in electrophysiologic assays of excitatory synapse number, strength, receptor composition, or presynaptic release probability at this stage (Figure 3).

The decrease in spine density appeared to result from a delay in the acquisition of spines, as neuroligin-1 depleted cells continued to acquire new spines over the following week. However, spine density in knockdown cells still remained less than controls at 28 days post-mitosis (control = 2.1 ± 0.1 spines/μm, *n* = 9; shNL1 = 1.5 ± 0.2 spines/μm, *n* = 8; *p* < 0.02). An analysis of spine morphology at this later stage demonstrated a shift toward larger spines in the control cells as would be expected during the maturation of adult-born granule cells (Toni et al., 2007; Zhao and Overstreet-Wadiche, 2008; Figure 2C). We also note that the reduction in spine density by neuroligin-1 knockdown that is no longer specifically confined to smaller filopodial spines, but is distributed across the range of spine volumes (Figure 2C).

### Neuroligin-1 knockdown delays dendritic outgrowth of adult-born granule cells

**Figure 4:**
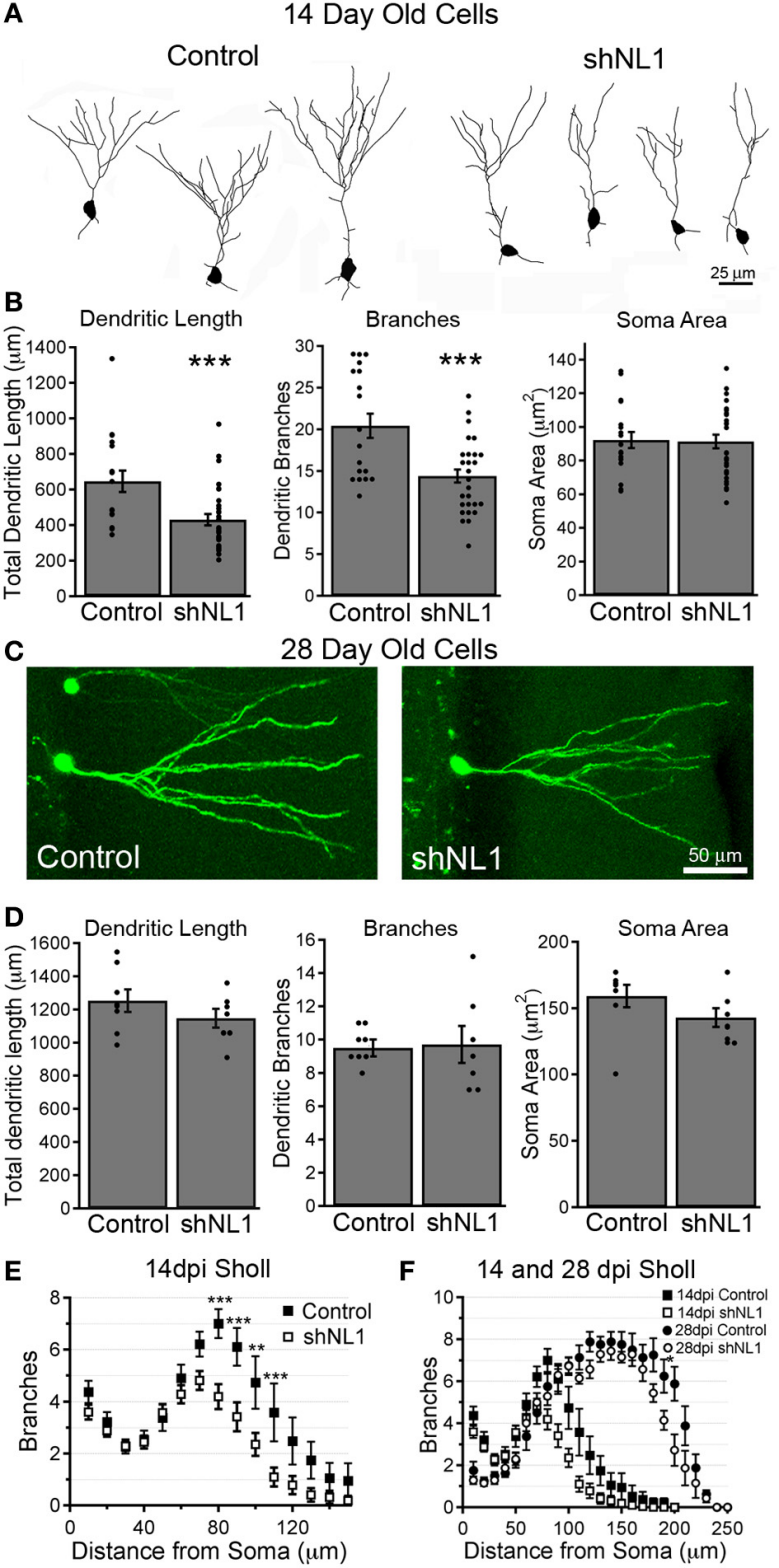
**Neuroligin-1 knockdown reduces dendritic complexity during early granule cell maturation. (A)** Representative dendrite branching patterns traced from control and neuroligin-1 knockdown (shNL1) newborn granule cells, 14 days post-mitosis. **(B)** Summary data of average soma cross-sectional area, dendritic branch number, and total dendritic length for 14-day-old control and neuroligin-1 knockdown cells (control, *n* = 19; shNL1, *n* = 32 cells; ^***^p < 0.001). **(C)** Representative newborn control and neuroligin-1 knockdown granule cells at 28 days post-mitosis. **(D)** Summary data of average soma cross-sectional area, dendritic branch number, and total dendritic length in 28 day-old cells. (control, *n* = 8; shNL1, *n* = 7 cells; *p* > 0.1). **(E)** Sholl analysis of dendritic branching of 14-day-old cells, demonstrating that neuroligin-1 knockdown decreases dendritic tree complexity (^**^p < 0.01; ^***^p < 0.001). **(F)** Sholl analysis of dendritic branching patterns of 28-day-old adult-born neurons, overlaid onto the Sholl plot for 14-day-old cells (^*^p < 0.05).

Because neuroligin-1 overexpression increases the formation of dendritic branches in immature, adult-born granule cells (Schnell et al., 2012), we examined the morphology of neuroligin-1 depleted cells at 14 days post-mitosis, during a stage of active dendritic outgrowth. Neuroligin-1 knockdown decreased the number of dendritic branches and total dendritic length at this stage (Figures 4A,B; *p* < 0.001). The decreased branching was confined to the inner molecular layer (IML), where dendrites first come in contact with excitatory afferents (Figure 4E). In contrast, at 28 days post-mitosis, when granule cells typically have a mature dendritic arbor, adult-born neurons in both groups had similar branch numbers and total dendritic length (Figures 4C,D; *p* > 0.4). Likewise, at 28 days the decreased dendritic branching in the IML was no longer apparent in the neuroligin-1 knockdown group, leaving only a subtle difference at the most distal extent of the dendritic tree (Figure 4F). Thus, neuroligin-1 knockdown delayed, but did not prevent, normal dendritic outgrowth.

### Neuroligin-1 knockdown reduces newborn granule cell survival

**Figure 5:**
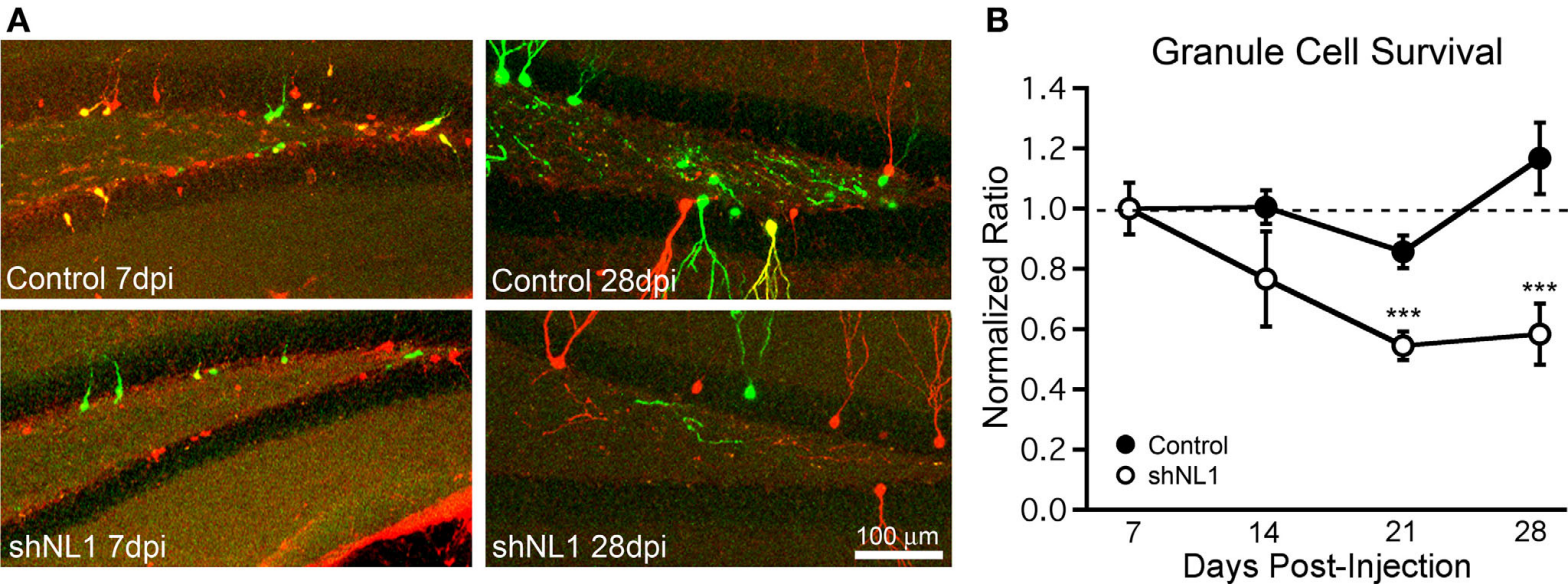
**Neuroligin-1 knockdown reduces newborn granule cell survival. (A)** Representative images of dentate gyrus, containing retrovirus-labeled newborn cells from co-injection survival experiments. Equal volumes of two viruses (GFP- and mCherry-coding) were injected, and animals were perfused at varying timepoints after injection. In control animals, GFP viruses were vector controls. In shNL1 animals, GFP viruses contained an shRNA against neuroligin-1. At 7 dpi (left panels), cell somata were smaller, predominantly located at the subgranular zone with rudimentary dendrites. GFP+ and mCherry+ tracks are overlaid; cells infected with both viruses are shown in yellow. Mice analyzed at later timepoints had a decreased ratio of GFP:mCherry cells in the neuroligin-1 knockdown group. **(B)** Summary graph demonstrating that neuroligin-1 knockdown decreases cell survival. The ratio of GFP+ to mCherry+ cells was obtained for each animal at 7 days, and subsequent ratios were normalized to this for each group independently. The ratio of GFP+ to mCherry+ cells decreased over time for shNL1 co-injected animals (shNL1 *n* = 7,8,7,5 animals; ^***^p < 0.005 vs. 7 days) but remained unchanged for control injections (control *n* = 7,6,5,6 animals; *p* > 0.2 each vs. 7 days).

Prior studies suggest the existence of a critical period for newborn granule cell survival between 2 and 3 weeks post-mitosis (Kempermann et al., 2003; Tashiro et al., 2006), coinciding with the onset of spine formation. Thus, we investigated whether neuroligin-1 knockdown altered survival of newborn granule cells using a retroviral co-injection strategy (Tashiro et al., 2006). In control animals, we co-injected an equal mixture of two viruses containing the genes for GFP and mCherry, respectively, which allowed us to control for variability in injection site accuracy and rates of neurogenesis between animals. The ratio of GFP:mCherry expressing cells at different timepoints was normalized to the ratio at 7 days post injection, which controlled for any differences in viral load. In control animals, the absolute number of retrovirus-labeled cells decreased over time as expected, such that the number of cells at 28 dpi was 44% of the number at 7 dpi (GFP+ cells per 100 μm section: 7 dpi = 24.8 ± 3.7, 14 dpi = 19.7 ± 1.3, 21 dpi = 13.8 ± 0.9, 28 dpi = 10.8 ± 1.4; *n* = 5–7 animals per group). However, the ratio of GFP:mCherry labeled cells remained constant over time (Figure 5B, solid markers; *p* > 0.2 for each time point vs. 7 dpi), indicating a similar survival of newborn cells infected with either control virus. However, in the neuroligin-1 knockdown condition, the ratio of GFP+ cells (containing the neuroligin-1 shRNA) to mCherry+ (control) cells decreased over time (Figure 5; *p* < 0.005 for both 21 and 28 dpi vs. 7 dpi). Thus, the reduction in neuroligin-1 substantially decreased the survival of newborn granule cells.

As GFP+ and mCherry+ cells were counted independently without regards to colocalization, any double-infected cells were counted in both groups. In a separate analysis of the mCherry + shNL1 condition at 7 dpi, mCherry+ cells were evaluated for co-infection with shNL1 virus. Only 7.1% ± 2.0% (*n* = 7 animals) of the mCherry+ cells were also GFP+ (and expressing the NL1 shRNA) at 7 dpi in this condition, leaving ~93% of mCherry cells as proper controls. However, as this small population of double-infected cells would only tend to decrease the magnitude of the observed survival difference between the groups, the effect of neuroligin-1 knockdown on survival could not have resulted from this small population of double-infected neurons.

## Discussion

### Neuroligin-1 involvement in granule cell morphologic development

Most analyses of the synaptogenic role of neuroligin-1 have focused on the transynaptic signaling/adhesion of neuroligin-1 with presynaptic neurexins, and on the ability of neuroligin-1 to recruit post-synaptic signaling complexes to excitatory synapses (Craig and Kang, 2007; Sudhof, 2008). However, our data suggest a more fundamental role for neuroligin-1 in dendritic branch and spine filopodium formation. Prior work has shown that neuroligin-1 stabilizes post-synaptic filopodia (Arstikaitis et al., 2011), and can even enhance filopodium formation in response to glutamate uncaging in the absence of a presynaptic contact (Kwon et al., 2012). In live imaging of *Xenopus* tadpoles, neuroligin-1 stabilized dendritic filopodia through interactions with extracellular neurexin proteins, and contributed to dendritic growth via a synaptotropic mechanism (Chen et al., 2010). Because filopodia are critical precursors to both spines (Ziv and Smith, 1996) and dendrites (Dailey and Smith, 1996), these data suggest a general role for neuroligin-1 in filopodial dynamics prior to both dendrite and spine formation.

### Newborn granule cell survival signaling

Our most dramatic finding was that neuroligin-1 knockdown reduced neuronal survival without overt changes in synaptic function. The reduction in survival became evident between the 2nd and 3rd post-mitotic week, which precedes the majority of excitatory synaptic innervation (Esposito et al., 2005; Zhao et al., 2006), but during a time window when our results indicate that neuroligin-1 is necessary for proper morphologic development.

What mechanisms could link neuroligin-1's effects on cell morphology with a role in cell survival signaling? Neuroligin-1 recruits synaptic proteins, including PSD-95 (Irie et al., 1997), to nascent synapses, which could directly organize a signaling cascade coupling spine assembly to anti-apoptotic mechanisms. For example, the Rho family GTPase Rac1, which requires PSD-95 to assemble a functional signaling complex (Choi et al., 2005), has a role in spine morphogenesis and dendrite growth (Vadodaria and Jessberger, 2013) and also promotes neuronal cell survival during adult neurogenesis (Haditsch et al., 2013).

Alternatively, neuroligin-1 might increase cell survival indirectly through its downstream effects. For example, neuroligin-1 dependent dendritic outgrowth may expose immature cells to more survival-promoting trophic factors, such as BDNF (Sairanen et al., 2005). Finally, synaptic function might have been subtly altered in ways not detected by our experiments, leading to changes in neuronal survival secondary to altered synaptic signaling (Tashiro et al., 2006). Further studies are necessary to distinguish between a survival-enhancing effect specific to neuroligin-1, from one that is mediated by proper morphologic development of immature neurons.

### Functional excitatory synapse formation by newborn granule cells

Prior studies of neuroligin knockdown have reported decreases in both dendritic spine density and excitatory synaptic currents in more mature preparations, both *in vitro* and *in vivo* (Chih et al., 2005; Shipman et al., 2011; Kwon et al., 2012; Shipman and Nicoll, 2012). Unlike mature neurons in which excitatory synapses are predominantly located at the tips of dendritic spines, immature adult-born granule cells contain substantial numbers of axodendritic synapses in addition to axospinous synapses (Toni et al., 2007). As many dendritic protrusions from immature granule cells also lack presynaptic terminals (Toni et al., 2007), neuroligin-1 knockdown might specifically have decreased the number of immature filopodia in immature cells. As neuroligin-1 is also involved in activity-dependent filopodium formation (Kwon et al., 2012), this may point to a role for neuroligin-1 in dendritic process outgrowth prior to the establishment of a functional synapse.

Because neuroligin-1 overexpression in immature granule cells increases excitatory synaptic function at 21 days post-mitosis (Schnell et al., 2012), one might have expected a decrease following neuroligin-1 knockdown. There are several possible explanations for this apparent discrepancy. First, if neuroligin-1 is preferentially involved in dendritic filopodium formation, it might function upstream of functional excitatory synapse formation onto dendritic spine synapses, yet not have a role at dendritic shaft synapses. In fact, immature granule cells have a substantial number of excitatory shaft synapses as well as many non-synaptic dendritic spine filopodia (Toni et al., 2007). In mature hippocampal granule cells, neuroligin-1 knockdown reduces dendritic spine density as well as excitatory synaptic currents (Shipman and Nicoll, 2012), which is consistent with a decrease in filopodium formation at an early stage leading to a decrease in functional excitatory synapses on dendritic spines at a later stage. Secondly, because neuroligin family members can compensate for one another (Varoqueaux et al., 2006; Budreck and Scheiffele, 2007), it is also possible that the role of neuroligin-1 in functional synapse formation (assayed electrophysiologically) is compensated by other neuroligins. In either case, the morphologic and survival phenotype appears to be more sensitive to neuroligin-1 knockdown than standard functional assays of the strength and receptor composition of excitatory synaptic innervation.

## Author contributions

Eric Schnell and Gary L. Westbrook conceived and designed the experiments. Eric K. Washburn designed and created reagents for the experiments. Eric Schnell, Thomas H. Long, and AeSoon L. Bensen performed experiments and analyzed data. Eric Schnell, Thomas H. Long, Eric K. Washburn, AeSoon L. Bensen, and Gary L. Westbrook wrote and edited the manuscript.

### Conflict of interest statement

The authors declare that the research was conducted in the absence of any commercial or financial relationships that could be construed as a potential conflict of interest.

## References

[B1] Ambrogini P. Cuppini R. Lattanzi D. Ciuffoli S. Frontini A. Fanelli M. (2010). Synaptogenesis in adult-generated hippocampal granule cells is affected by behavioral experiences. Hippocampus 20, 799–810 10.1002/hipo.20679 19623538

[B2] Arstikaitis P. Gauthier-Campbell C. Huang K. El-Husseini A. Murphy T. H. (2011). Proteins that promote filopodia stability, but not number, lead to more axonal-dendritic contacts. PLoS ONE 6:e16998 10.1371/journal.pone.0016998 21408225 PMC3049770

[B3] Budreck E. C. Scheiffele P. (2007). Neuroligin-3 is a neuronal adhesion protein at GABAergic and glutamatergic synapses. Eur. J. Neurosci. 26, 1738–1748 10.1111/j.1460-9568.2007.05842.x 17897391

[B4] Chen S. X. Tari P. K. She K. Haas K. (2010). Neurexin-neuroligin cell adhesion complexes contribute to synaptotropic dendritogenesis via growth stabilization mechanisms *in vivo*. Neuron 67, 967–983 10.1016/j.neuron.2010.08.016 20869594

[B5] Chih B. Engelman H. Scheiffele P. (2005). Control of excitatory and inhibitory synapse formation by neuroligins. Science 307, 1324–1328 10.1126/science.1107470 15681343

[B6] Choi J. Ko J. Racz B. Burette A. Lee J. R. Kim S. (2005). Regulation of dendritic spine morphogenesis by insulin receptor substrate 53, a downstream effector of Rac1 and Cdc42 small GTPases. J. Neurosci. 25, 869–879 10.1523/JNEUROSCI.3212-04.2005 15673667 PMC6725612

[B7] Craig A. M. Kang Y. (2007). Neurexin-neuroligin signaling in synapse development. Curr. Opin. Neurobiol. 17, 43–52 10.1016/j.conb.2007.01.011 17275284 PMC2820508

[B8] Dailey M. E. Smith S. J. (1996). The dynamics of dendritic structure in developing hippocampal slices. J. Neurosci. 16, 2983–2994 8622128 10.1523/JNEUROSCI.16-09-02983.1996 PMC6579052

[B9] Engert F. Bonhoeffer T. (1999). Dendritic spine changes associated with hippocampal long-term synaptic plasticity. Nature 399, 66–70 10.1038/19978 10331391

[B10] Esposito M. S. Piatti V. C. Laplagne D. A. Morgenstern N. A. Ferrari C. C. Pitossi F. J. (2005). Neuronal differentiation in the adult hippocampus recapitulates embryonic development. J. Neurosci. 25, 10074–10086 10.1523/JNEUROSCI.3114-05.2005 16267214 PMC6725804

[B11] Gould E. Beylin A. Tanapat P. Reeves A. Shors T. J. (1999). Learning enhances adult neurogenesis in the hippocampal formation. Nat. Neurosci. 2, 260–265 10.1038/6365 10195219

[B12] Haditsch U. Anderson M. P. Freewoman J. Cord B. Babu H. Brakebusch C. (2013). Neuronal rac1 is required for learning-evoked neurogenesis. J. Neurosci. 33, 12229–12241 10.1523/JNEUROSCI.2939-12.2013 23884931 PMC3721836

[B13] Irie M. Hata Y. Takeuchi M. Ichtchenko K. Toyoda A. Hirao K. (1997). Binding of neuroligins to PSD-95. Science 277, 1511–1515 10.1126/science.277.5331.1511 9278515

[B14] Kempermann G. Gast D. Kronenberg G. Yamaguchi M. Gage F. H. (2003). Early determination and long-term persistence of adult-generated new neurons in the hippocampus of mice. Development 130, 391–399 10.1242/dev.00203 12466205

[B15] Kempermann G. Kuhn H. G. Gage F. H. (1997). More hippocampal neurons in adult mice living in an enriched environment. Nature 386, 493–495 10.1038/386493a0 9087407

[B16] Kwon H. B. Kozorovitskiy Y. Oh W. J. Peixoto R. T. Akhtar N. Saulnier J. L. (2012). Neuroligin-1-dependent competition regulates cortical synaptogenesis and synapse number. Nat. Neurosci. 15, 1667–1674 10.1038/nn.3256 23143522 PMC3536444

[B17] Luikart B. W. Bensen A. L. Washburn E. K. Perederiy J. V. Su K. G. Li Y. (2011). miR-132 mediates the integration of newborn neurons into the adult dentate gyrus. PLoS ONE 6:e19077 10.1371/journal.pone.0019077 21611182 PMC3096628

[B18] Ming G. L. Song H. (2011). Adult neurogenesis in the mammalian brain: significant answers and significant questions. Neuron 70, 687–702 10.1016/j.neuron.2011.05.001 21609825 PMC3106107

[B19] Sairanen M. Lucas G. Ernfors P. Castren M. Castren E. (2005). Brain-derived neurotrophic factor and antidepressant drugs have different but coordinated effects on neuronal turnover, proliferation, and survival in the adult dentate gyrus. J. Neurosci. 25, 1089–1094 10.1523/JNEUROSCI.3741-04.2005 15689544 PMC6725966

[B20] Schnell E. Bensen A. L. Washburn E. K. Westbrook G. L. (2012). Neuroligin-1 overexpression in newborn granule cells *in vivo*. PLoS ONE 7:e48045 10.1371/journal.pone.0048045 23110172 PMC3478279

[B21] Shipman S. L. Nicoll R. A. (2012). A subtype-specific function for the extracellular domain of neuroligin 1 in hippocampal LTP. Neuron 76, 309–316 10.1016/j.neuron.2012.07.024 23083734 PMC3998838

[B22] Shipman S. L. Schnell E. Hirai T. Chen B. S. Roche K. W. Nicoll R. A. (2011). Functional dependence of neuroligin on a new non-PDZ intracellular domain. Nat. Neurosci. 14, 718–726 10.1038/nn.2825 21532576 PMC3171182

[B23] Sierra A. Encinas J. M. Deudero J. J. Chancey J. H. Enikolopov G. Overstreet-Wadiche L. S. (2010). Microglia shape adult hippocampal neurogenesis through apoptosis-coupled phagocytosis. Cell Stem Cell 7, 483–495 10.1016/j.stem.2010.08.014 20887954 PMC4008496

[B24] Sudhof T. C. (2008). Neuroligins and neurexins link synaptic function to cognitive disease. Nature 455, 903–911 10.1038/nature07456 18923512 PMC2673233

[B25] Tashiro A. Sandler V. M. Toni N. Zhao C. Gage F. H. (2006). NMDA-receptor-mediated, cell-specific integration of new neurons in adult dentate gyrus. Nature 442, 929–933 10.1038/nature05028 16906136

[B26] Taupin P. Gage F. H. (2002). Adult neurogenesis and neural stem cells of the central nervous system in mammals. J. Neurosci. Res. 69, 745–749 10.1002/jnr.10378 12205667

[B27] Toni N. Teng E. M. Bushong E. A. Aimone J. B. Zhao C. Consiglio A. (2007). Synapse formation on neurons born in the adult hippocampus. Nat. Neurosci. 10, 727–734 10.1038/nn1908 17486101

[B28] Tronel S. Fabre A. Charrier V. Oliet S. H. Gage F. H. Abrous D. N. (2010). Spatial learning sculpts the dendritic arbor of adult-born hippocampal neurons. Proc. Natl. Acad. Sci. U.S.A. 107, 7963–7968 10.1073/pnas.0914613107 20375283 PMC2867872

[B29] Vadodaria K. C. Jessberger S. (2013). Maturation and integration of adult born hippocampal neurons: signal convergence onto small Rho GTPases. Front. Synaptic Neurosci. 5:4 10.3389/fnsyn.2013.00004 23986696 PMC3752586

[B30] van Praag H. Kempermann G. Gage F. H. (2000). Neural consequences of environmental enrichment. Nat. Rev. Neurosci. 1, 191–198 10.1038/35044558 11257907

[B31] van Praag H. Schinder A. F. Christie B. R. Toni N. Palmer T. D. Gage F. H. (2002). Functional neurogenesis in the adult hippocampus. Nature 415, 1030–1034 10.1038/4151030a 11875571 PMC9284568

[B32] Varoqueaux F. Aramuni G. Rawson R. L. Mohrmann R. Missler M. Gottmann K. (2006). Neuroligins determine synapse maturation and function. Neuron 51, 741–754 10.1016/j.neuron.2006.09.003 16982420

[B33] Zhao C. Teng E. M. Summers R. G. Jr. Ming G. L. Gage F. H. (2006). Distinct morphological stages of dentate granule neuron maturation in the adult mouse hippocampus. J. Neurosci. 26, 3–11 10.1523/JNEUROSCI.3648-05.2006 16399667 PMC6674324

[B34] Zhao C. S. Overstreet-Wadiche L. (2008). Integration of adult generated neurons during epileptogenesis. Epilepsia 49(Suppl. 5), 3–12 10.1111/j.1528-1167.2008.01632.x 18522595

[B35] Ziv N. E. Smith S. J. (1996). Evidence for a role of dendritic filopodia in synaptogenesis and spine formation. Neuron 17, 91–102 10.1016/S0896-6273(00)80283-4 8755481

[B36] Zucker R. S. Regehr W. G. (2002). Short-term synaptic plasticity. Annu. Rev. Physiol. 64, 355–405 10.1146/annurev.physiol.64.092501.114547 11826273

